# Supplementary material for: Elucidating optical field directed hierarchical self-assembly of homogenous versus heterogeneous nanoclusters with femtosecond optical tweezers
Source: PLoS One. 2019 Oct 31;14(10):e0223688. doi: 10.1371/journal.pone.0223688 (PMC6822744; doi:10.1371/journal.pone.0223688)
Supplement: Attachment — Submitted filename: Reply to reviewer 2.docx [file pone.0223688.s003.docx]

Reviewer #2: Predication of morphology is very important to design and facilitate materials with high performance properties. The authors stated they developed a theoretical method to predict morphologies of nano-clusters by solving the Langevin equation. It is a meaningful topic of the nano-research, and the present paper would have a wide interest of readers. It is suitable for the journal, PLOS ONE. However, there are several key problems to be clarified before accepting.

We are happy that the reviewer likes our work. We also would like to thank the reviewer for his encouraging comments and wonderful suggestion for the improvement of the manuscript. In our revised manuscript, all specific comments raised by the reviewers have been addressed below. We have highlighted the revised version of the manuscript to address all the issues raised by the reviewers.

1. Section Results and Discussion, the authors list Langevin equation (i.e. equation 1), and the equation derived from Langevin equation. They stated “By solving the above equation, we can fit our experimental one-sided power spectrum into theoretical power spectrum”. It lacks of necessary process for derivation. Because the authors stated they provide a model, and equation 2 is the governing equation of the model. It at least refers to the model. Please provide more details on the derivation from equation 1 to 2.

We appreciate the reviewer for detail concern of our data analysis method. We have now provided below the detailed derivation from equation 1 to 2 and which can also be found in reference 16 (K. Berg-Sørensen and H. Flyvbjerg, Rev. Sci. Instrum. 75,594(2004).) in the main manuscript.

According to Einstein-Ornstein-Uhlenbeck theory of Brownian motion, the position fluctuation of a particle trapped in a harmonic potential is given by the modified Langevin equation:

$m\ddot{x}\left( t \right)+\gamma_{0}\dot{x}\left( t \right)+\kappa x\left( t \right)= \sqrt{{2k}_{B}T\gamma_{0}} \zeta(t)$--------------- (1)

Here x(t) is the time-dependent trajectory of the particle. m and γ_0_ are the mass and friction coefficient of the particle, respectively. The force due to the harmonic nature of the optical trap can be considered as –κx(t), where κ is the spring constant or trap stiffness.

Stokes law for a spherical particle of radius r states that the viscous drag coefficient is:

γ_0_ =6πrηρ ----------------- (2)

Here, η is the kinematic viscosity, ρ is the density of the medium. So, η×ρ is the fluid’s shear viscosity.

The right-hand side of the above equation 1 represents a random Gaussian process representing Brownian forces at a temperature T in absolute scale. The time-averaged function $\left\langle\zeta(t) \right\rangle$ is zero and $\left\langle\zeta\left( t \right)\zeta(t^{'}) \right\rangle=\delta(t-t^{'})$.

Equation (1) can be rewritten as:

$\frac{m}{\gamma_{0}}\ddot{x}\left( t \right)+\dot{x}\left( t \right)+\frac{\kappa}{\gamma_{0}} x\left( t \right)= \frac{\sqrt{{2k}_{B}T\gamma_{0}}}{\gamma_{0}} \zeta\left( t \right)$

Or, $\frac{m}{\gamma_{0}}\ddot{x}\left( t \right)+\dot{x}\left( t \right)+\frac{\kappa}{\gamma_{0}} x\left( t \right)= \sqrt{\frac{{2k}_{B}T}{\gamma_{0}}} \zeta\left( t \right)$

Now,$\frac{k_{B}T}{\gamma_{0}}=D$. This D is the diffusion coefficient. Now, the above equation becomes,

$\frac{m}{\gamma_{0}}\ddot{x}\left( t \right)+\dot{x}\left( t \right)+\frac{\kappa}{\gamma_{0}} x\left( t \right)= \sqrt{2D} \zeta\left( t \right)$ ---------------- (3)

The characteristic time for loss of kinetic energy through friction is m/γ_0_, which is in nanosecond timescale and well beyond our measuring capability through QPD detector (10μs resolution). Thus, the first term of equation 3 can be neglected in our experimental condition resulting in the following simplified equation:

$\dot{x}\left( t \right)+\frac{\kappa}{\gamma_{0}} x\left( t \right)= \sqrt{2D} \zeta\left( t \right)$ --------------- (4)

Now, if we define$\frac{\kappa}{\gamma_{0}}=2\pi f_{c}$, where *f*_c_ is defined as another constant, the corner frequency, then the above equation becomes:

$\dot{x}\left( t \right)+2\pi f_{c} x\left( t \right)= \sqrt{2D} \zeta\left( t \right)$ --------------------- (5)

In the experiment if the data is collected up to time t_0_, then the Fourier transform of the function x(t) can be done by:

$$\tilde{x_{k}}=\int_{\frac{-t_{0}}{2}}^{\frac{t_{0}}{2}} x\left( t \right)e^{-2\pi if_{k} t} dt$$

Here, *f*_k_ = k/t_0_ and k is an integer.

Using the Fourier transform, equation 5 yields:

$\tilde{x_{k}}=\frac{\sqrt{2D}}{2\pi\left( f_{c}-if_{k} \right)}\tilde{\zeta_{k}}$ ----------------- (6)

As the function ζ(t) is Gaussian in time domain, the Fourier transform of it $\tilde{\zeta}_{k}$ is also Gaussian in nature. So, the relations $\left\langle\tilde{\zeta_{k}} \right\rangle$ and $\left\langle\tilde{\zeta_{k}^{*}}\tilde{\zeta_{k}} \right\rangle={t_{0}\delta}_{k,l}$ holds. Power spectra (*P_x_*(*f*)) is defined as:

$$P_{x}\left( f \right)=\frac{\left| \tilde{x_{k}} \right|^{2}}{t_{0}}$$

Or, $P_{x}\left( f \right)=\frac{D\left| \tilde{\zeta_{k}} \right|^{2}}{t_{0}2\pi^{2}\left( f_{c}^{2}+f^{2} \right)}$

Or, $P_{x}\left( f \right)=\frac{D}{2\pi^{2}\left( f_{c}^{2}+f^{2} \right)}$

The above function does not distinguish between positive and negative frequencies. In this case, one sided power spectrum can be defined as:

$$P_{x}\left( f \right)=\frac{D}{\pi^{2}\left( f_{c}^{2}+f^{2} \right)}$$

2. In tables 1, 2 and 3, Why did the authors choose the values of the trapping laser power which are not the arithmetic sequences. Why are there the special values of 17.5, 22.5?

We acknowledge the point raised by the reviewer. In our experimental set up (Figure 1), a λ/2 waveplate in union with a polarizer beam splitter (PBS) controls the power of the input laser to the home built optical tweezers setup. The λ/2 waveplate is rotated resulting in rotation of light polarization. The fixed polarizing beam splitter allows a fraction of the laser power to pass according to its relative orientation of local axes. We measure the average laser power before the lens assembly L1-L2. After this, the beam traverses through the objective and loses laser power. Every day basis, we optimize our set up by trapping 1000 nm diameter bead without rotating waveplate angle. This power is measured 17.5 mW, and we always calibrate our set up with this power because we can achieve a long period of trapping. Otherwise, there is no specific intention to choose such power, and 22.5 mW power is chosen to have 5 mW power interval from the 17.5 mW.

Figure 1. Schematic diagram of our experimental femtosecond optical tweezers setup. WP: Half-wave plate; PBS: Polarizing beam splitter; L1: Concave lens; L2: collimating convex lens; DM: Dichroic mirror; O: Objective lens; SS: Sample stage; C: Condenser lens; GF: Green filter; L3: Focusing lens; QPD: Quadrant photodiode; SM: Silver mirror; RF: Red filter; CCD: Camera (Charge-coupled device) PC: Personal computer.

3. The text below figure 4, lines 1-3, “To further investigation into this”, what does “this” refer to? “This shows that at low power”, what is the mean of “this”? “the particles can be considered as non-interacting, and the theoretical equation derived before holds.” How could the “non-interacting” be concluded? Please explain more about it.

We thank the reviewer for critically arising these points. In the main manuscript, by “this”, we mean to investigate the generality of equation 4 in different trapping power. Also, the experiment is extended to the three-particle cluster formation analysis. The further experimental results prove that the equation 4 holds correct within the assumption of non-interacting bead scenario where the power spectrum of ‘N’ number of beads can simply be added linearly without involving any cross terms. We have shown below (Figure 2) how much experimental fitted data deviates from our theoretical model data. We have now rewritten those lines and clearly explained our motivation for performing power-dependent study for dimer as well as a trimer. We have highlighted that section in the revised version of the manuscript.

Figure 2. The comparison between experimental fitted data and theoretical model fitted line.

4. All the equations, please explain the physical meanings of every symbol. I failed to find the exact explanations of P_x, x^{\tilde}, f_c.

We understand the concern of the reviewer. We have now explained all the terms along with derivation in the response of reviewer query 1. P_x_ is power spectra, x^(/tilde) is the Fourier transform of the time-dependent position x(t), and fc is corner frequency. We have now explained every symbol that we have mentioned in the revised version of the manuscript.

5. Section Conclusion, line 2 from bottom, what does the “fluctuation-dissipation theorem” refer to in the present paper? Does it refer to Langevin equation or the equations corresponding to Langevin equation? The reviewer think that the data partly support such the conclusion.

We appreciate the concern of the reviewer regarding our conclusion part. Fluctuation Dissipation theorem provides a theoretical model for Brownian motion. The line mentioned in the reviewer’s comment points towards a plausible future direction of our work where we plan to utilize the model of Brownian motion of fluctuation-dissipation theorem and merge into the power spectrum method of data analysis.

We also agree that the data presented in this paper partly support such conclusion because we have not performed such an experiment with biomolecules. Therefore, we have removed this part in the revised version of the manuscript.

6. Line 3 below equation 1, kB should be k_B.

We are thankful to the reviewer for pointing out this our error. We have now corrected this mistake in the revised version of the manuscript and highlighted that part.
