# Supplementary material for: Elucidating optical field directed hierarchical self-assembly of homogenous versus heterogeneous nanoclusters with femtosecond optical tweezers
Source: PLoS One. 2019 Oct 31;14(10):e0223688. doi: 10.1371/journal.pone.0223688 (PMC6822744; doi:10.1371/journal.pone.0223688)
Supplement: Attachment — Submitted filename: Reply to reviewer 1.docx [file pone.0223688.s004.docx]

Please use the space provided to explain your answers to the questions above. You may also include additional comments for the author, including concerns about dual publication, research ethics, or publication ethics. (Please upload your review as an attachment if it exceeds 20,000 characters)

Reviewer #1: The author has provided detailed replies to all the comments. I am not sure if it is some conversion error. Equation 3 and 4 are still not showing in the correct form, while they were in correct form in the section of response to the comments. I urge the author to fix the appropriate typo errors.

We are sorry for this conversion error that skipped our attention. We thank the reviewer for detecting this and we have revised the manuscript to take care of this problem. We provide the revised manuscript.
